# Insights on physiological, antioxidant and flowering response to salinity stress of two candidate ornamental species: the native coastal geophytes *Pancratium maritimum* L. and *Eryngium maritimum* L

**DOI:** 10.1007/s12298-024-01502-0

**Published:** 2024-08-27

**Authors:** Marcello Dante Cerrato, Pere Miquel Mir-Rosselló, Iván Cortés-Fernández, Arnau Ribas-Serra, Cyril Douthe, Carles Cardona, Antoni Sureda, Jaume Flexas, Lorenzo Gil Vives

**Affiliations:** 1 Interdisciplinary Ecology Group, Department of Biology, University of the Balearic Islands, 07122 Palma, Balearic Islands Spain; 2 Research Group On Plant Biology Under Mediterranean Conditions, Departament de Biologia, Universitat de Les Illes Balears (UIB) -Agro-Environmental and Water Economics Institute (INAGEA), E-07122 Palma, Balearic Islands Spain; 3 Research Group in Community Nutrition and Oxidative Stress, University of the Balearic Islands- IUNICS, E-07122 Palma, Balearic Islands Spain; 4 CIBEROBN (Physiopathology of Obesity and Nutrition), Instituto de Salud Carlos III, E-28029 Madrid, Spain; 5 Health Research Institute of Balearic Islands (IdISBa), E-07120 Palma, Balearic Islands Spain; 6 Centre Forestal de les Illes Balears (CEFOR‑Menut), C/Gremi de Corredors 10 (Polígon Son Rossinyol), Institut Balear de la Natura (IBANAT), 07009 Palma, Spain

**Keywords:** Salinity, ROS, Ornamental, Coastal, Gas-exchange

## Abstract

Increasing seawater influence in coastal areas is an ongoing environmental issue. Gardening is a widespread activity mainly in touristic areas such as the Mediterranean coasts. However, the use of exotic species well adapted to salinity encompasses the risk of invasive species introduction. This study aimed to evaluate salinity tolerance of native geophytes, *Pancratium maritimum* L. and *Eryngium maritimum* L., to assess their use as ornamental species in salt affected coastal areas. Experiments were conducted using cultivated plants for flowering response and physiological and enzymatic antioxidant response. Six treatments were applied for two months, exposing plants to seawater (SW) dilutions (Tap-Water, 6.25%SW, 12.5%SW, 25%SW, 50%SW and 100%SW). Taxa decreased inflorescence production being this effect more architectonical in *E. maritimum* and affecting all inflorescence integrity in *P. maritimum*. Flowering time was strongly delayed and reduced in *P. maritimum*, while *E. maritimum* showed smaller effects among treatments. Physiological and biochemical response showed at moderate salinity levels (1/4SW) variation concomitant with late stress response and senescence in *P. maritimum,* with decreased water use efficiency, NPQ values, and enzymatic activity, and increased malondialdehyde (MDA) levels. In contrast, *E. maritimum* showed early stress response with steady gas exchange response, increasing NPQ values and catalase (CAT) and superoxide dimutase (SOD) activity, and decreasing MDA levels with salinity. Glutathione enzymes showed limited participation in both species. The results of this study suggest that neither species can be classified as halophytes, but they exhibit tolerance to low and moderate salinity levels, making them suitable for ornamental use.

**Supplementary Information:**

The online version contains supplementary material available at 10.1007/s12298-024-01502-0.

## Introduction

Coastal areas face escalating salinity from storms and flooding (Maun 2009; Cozzolino et al. 2017; Du and Hesp 2020), expected to worsen with rising sea levels, seawater intrusion, and higher storm frequency (IPCC 2014; Rizzetto 2020). These factors entail important threats to vegetation particularly in densely populated coastal destinations worldwide (González-Baheza and Arizpe 2018; Pulido-Bosch et al. 2019; Rizzetto 2020). In the Mediterranean, urban growth and gardening enhance landscapes for tourism (Heywood 2017), with re-vegetation efforts being dual for coastal protection against seawater and erosion while also for touristic appeal (Semeoshenkova & Newton 2015).

Salinity greatly influences coastal plant selection for thriving growth and appealing flowering (Ferrante et al. 2011). Stress factors like salinity compromise plant development (Cassaniti et al. 2012; 2013; García-Caparrós & Lao 2018), often showing negative correlations between soil salinity and flowering vigor (Cassaniti et al. 2013). Salt deleterious effects are mainly derived from two major processes which are the osmotic effect -capacity to absorb water- and the ionic effect -toxicity derived from Na^+^ and Cl^−^ absorption- (Munns and Tester 2008). Both processes lead to stress conditions defined by a context of water deficits, impaired photosynthesis, reactive oxygen species (ROS) production, and photochemical damage (Flexas et al. 2004, 2006; Arora et al. 2016). Plants respond with physiological (stomatal regulation, metabolic adjustments), morphological (growth cycle, leaf structure changes), and biochemical mechanisms (antioxidants like SOD, CAT, GPx, AsA-GSH cycle) (Galmés et al. 2007; Sharma et al. 2012; Gupta et al. 2016).

For coastal gardening, selecting species resilient to salinity and drought is crucial (Yasheshwar et al. 2017; Atzori et al. 2019). Many species show ornamental potential under saline conditions (Cassaniti et al. 2012; 2013; García-Caparrós and Lao 2018; Guo et al. 2022; Piccolo et al. 2023), but careful trait selection is needed to prevent invasive species introduction (Pyšek et al. 2011; van Kleunen et al. 2018), especially in the Mediterranean (Cerrato et al. 2023). Native taxa have been suggested as an alternative for modern landscape gardening, and specifically, for the Mediterranean regions this idea has gained support considering the varied and well adapted flora (Fascetti et al. 2014; Krigas et al. 2021; Leotta et al. 2023). Among these taxa, geophytes (plants with bulbs or rhizomes) have been of special interest since they combine desirable traits such as abundant flowering, ease of transportation and cultivation, and resilience to stress conditions (Fascetti et al. 2014; Vicedo et al. 2021).

Studies on salinity's effect on native ornamentals are limited, with a focus on ecological rather than reproductive traits. *Pancratium maritimum* L. and *Eryngium maritimum* L. are potential ornamentals, known for vegetative reproduction, attractive inflorescence (Cassaniti et al. 2013; Paradiso et al. 2009), and an extensive distribution covering Mediterranean and Atlantic coast (Medrano et al. 1999; De Castro et al. 2020; Isermann & Rooney 2014). Both species tolerate high salinity (Meot-Duros et al. 2008; Ivanova et al. 2015; Mohamed et al. 2018). However, specific response to soil salinity has only been partially assessed on vegetative traits for *P. maritimum* (Khedr et al. 2003; Carfagna et al. 2021) and reproductive response in *E. maritimum* (Cortés-Fernández et al. 2022a, b).

Promoting these species aids in conserving declining native populations due to habitat loss (Grassi et al. 2005; Necajeva and Ievinsh 2013), supporting local ecosystems and pollinators (Garbuzov et al. 2017). Researching their potential as conservation tools and alternatives to invasive species is vital.

This study evaluates salinity’s impact on *P. maritimum* L. and *E. maritimum* L., exploring their potential as ornamentals in saline coastal areas. Objectives include:

1.Assessing physiological and antioxidant responses to salinity stress.

2.Evaluating reproductive responses to salinity stress.

3.Examining implications for their development and ornamental use under salinity stress.

## Material and methods

### Plant material and experimental design

**Fig. 1:**
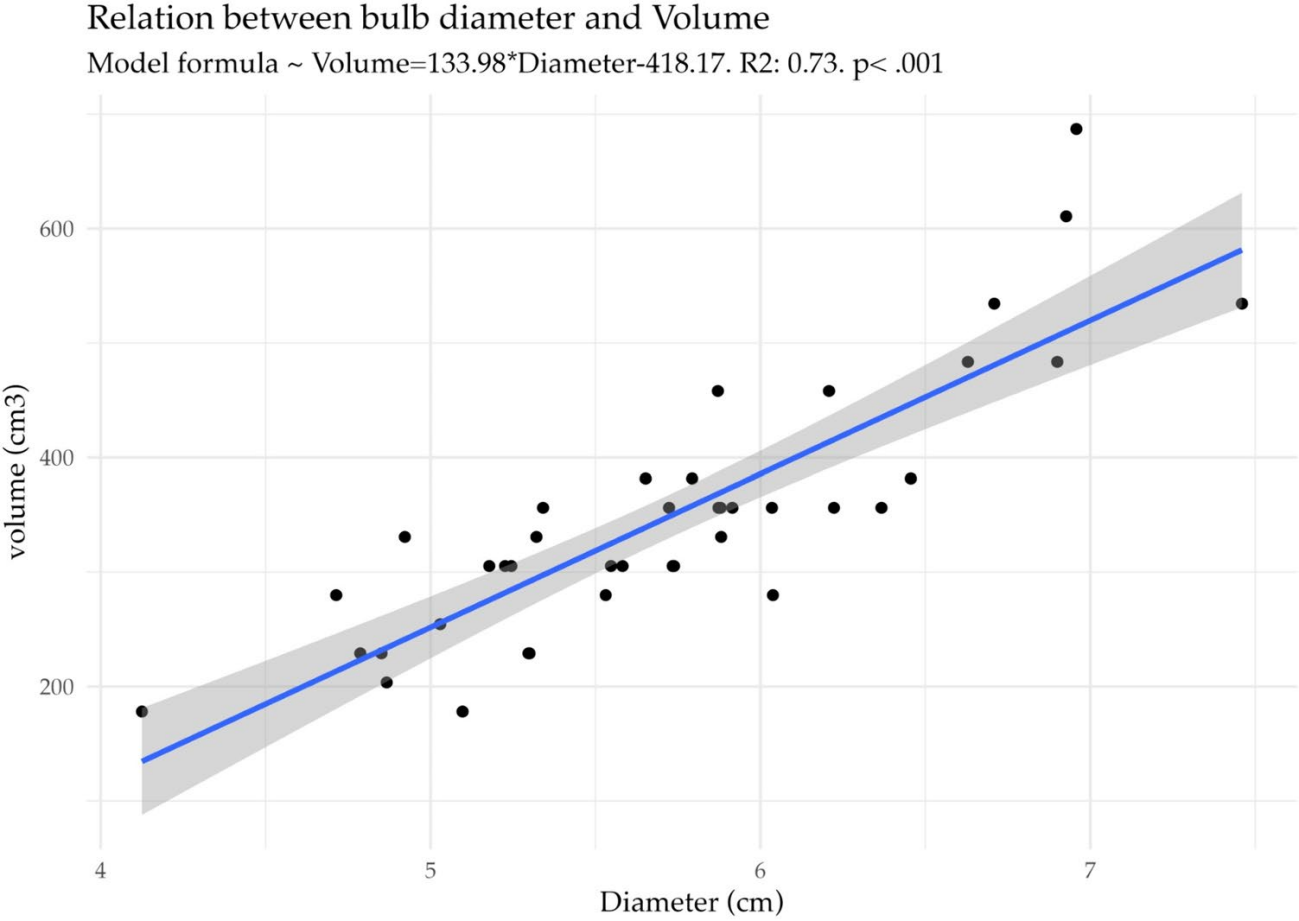
Bulb diameter relation to bulb volume

For plant production in both experiments (physiological-antioxidant response and reproductive response) seeds were collected from Son Serra de Marina (Blinded). For reproductive response, 60 bulbs of *P. maritimum* were collected near the urban area of Son Serra de Marina (Blinded) in August 2019 (Permit: Blinded). Criteria of the collection were size and ease of collection to minimize damage to the bulb and impact on the surrounding area. Size selection followed the procedure described by Gil (1994) where minimum bulb volume is established for flowering. Since there is a high relation between diameter and volume (Fig. 1) bulbs were selected and evenly distributed among treatments according to diameter. Two experiments were conducted using plant material as follows.

- Experiment 1 (Physiological-Antioxidant response): Plant production using seeds germinated at 20 °C with the seed of *E. maritimum* undergoing cold stratification, as indicated by Cortés-Fernández et al. (2021), prior to germination essay. The growth period of *P. maritimum* plantlets was grown for 1 year and 5 months to ensure sufficient leaf development for gas exchange and antioxidant measurements, due to their slower growth rate. *E. maritimum* plantlets were grown for 5 months prior to the experiment trial. Pot size for both species was 3-L pots throughout the experiment. Treatment application was applied in March 2021. Measurement timing was taken two months after treatment application, in May 2021. Measurements: Gas exchange and fluorescence measurements and leaf collection for antioxidant and MDA levels analysis.
- Experiment 2 (Reproductive Response): Plant production using bulbs collected from Son Serra and randomly allocated to different treatments. Plants were reallocated based on measurements to avoid size bias. For *E. maritimum* two-year-old plants grown from seed under equal conditions as Experiment 1 plants (Cortés-Fernández et al. 2022a, b). The growth period of *P. maritimum* comprised the bulbs collected and cultivated in same conditions for 5 months prior to treatment application, while *E. maritimum* comprised plant from seeds maintained during 2 years in 3 L-pots until 5 months prior to treatment application (transplanted to 5 L-pots). Pot size for both species was 5-L pots throughout the experiment. Treatment application was applied in March 2020 for *P. maritimum* before leaf senescence in June, and mid-May for *E. maritimum* (two months prior to flowering). Measurement timing occurred when plants flowered in July. Measurements were flowering traits.

### Growing conditions and treatment application

For each experiment, 60 plants per species (a total of 120 plants per species) were allocated to six seawater treatments based on electric conductivity (EC in dS/m): Control-Tap Water (1.05), 6.25% SW (5.07), 12.5% SW (9.30), 25% SW (16.34), 50% SW (30.30), and 100% SW (55.69). Each treatment was applied as described in Cerrato et al. (2022) and Cortés-Fernández et al. (2022a, b), with one month of pre-treatment followed by two months of full treatment.

Considering plant size and limited pot volume, a culture substrate was selected to ensure plant growth and proper reproductive development. To avoid cultivation limitations as observed in similar studies (Pujenwoeck et al. 2017) we opted for the following media. The substrate was composed of 61.50% coconut fiber, 33.00% white peat moss, and 5.50% expanded perlite, with fertilization using 4.40 mg/ l of Osmocote NPK 19–10-19, a slow-release fertilizer. The experiments took place outdoors under a shade cloth that excluded 50% of the light. Seawater was collected from Sa Ràpita locality and stored for its use at the beginning of the experiment following the storage recommendation by Hanley et al. (2020). Treatments were applied by combining the proportion of seawater with tap-water of the University facility to fulfill each of the six treatments (including control) mentioned above. Watering was done until field capacity with variable frequency depending on soil moisture (based on pot weight relative to maximum weight) ranging from weekly to three times per week. Soil conductivity was periodically measured (XS Instruments Cond 51 +) to avoid excess salinity (higher conductivity values than the corresponding treatment, see above) accumulating in the substrate. Soil conductivity was assessed according to Shahid et al. (2018). Soil samples were diluted in distilled water at a 1:5 ratio and mixed with a magnetic shaker for 2 h. After filtration, conductivity was measured using an XS Instruments Cond 51 + device. If any variation was detected, a watering event with tap water was conducted accompanied with subsequent treatment application.

### Reproductive measurements

Reproductive traits measured in *E. maritimum* are described in Cortés-Fernández et al. (2022a, b). In short, traits measured in the present study were inflorescence length and diameter, number of capitula of each scapus, length and width of first and second whorls, and fruit and seedset. Further details can be found in the previous reference.

For *P. maritimum* inflorescence measurements were conducted using the total length and diameter of flowers and the stem. Each flower produced per plant was counted and measured both in length and width using external tepals. Additionally, further specific measurements were conducted but provided as supplementary data (Supplementary data Table S1). Fruit set was determined by calculating the ratio of the number of fruits produced to the number of flowers per plant. Seed set was calculated based on the number of seeds produced per fruit, using the average number of seminal primordia determined from randomly selected and dissected flowers in each treatment.

Phenological data was recorded in both species. In *E. maritimum* the number of whorls in the flowering stage was recorded each week. For *P. maritimum*, since the flowering time of each of the flower lasts just one day (Gil 1994), the number of flowers per plant was recorded each day. The sum of flowers per plant each week was used for joined analysis with *E. maritimum*.

### Gas exchange and fluorescence measurements

Two months after subjecting the plants to full salinity treatments, gas-exchange measurements were conducted for each species. Each treatment group (N = 10 plants) underwent measurements using an open gas-exchange system equipped with a 2 cm^2^ fluorescence chamber (Li-6400, Li-cor Inc., Lincoln, USA). To prevent bias from the time of day, species and treatments were randomly selected for measurement between 10:00 and 14:00, with adjustments made for humidity and temperature based on environmental conditions. Light saturation was maintained at approximately 1500 µmol m^−2^ s^−1^, with a CO_2_ concentration of 400 µmol mol^−1^ and a flow rate of 300 µmol s^−1^. For *E. maritimum*, leaf area correction was unnecessary as the leaves adequately covered the chamber. For *P. maritimum*, digital images were captured and analyzed using Fiji software (Schindelin et al. 2012) to correct for leaf area variations. The parameters measured included net assimilation rate (A*n*), stomatal conductance (gs), intercellular CO_2_ concentration (Ci), and transpiration rate (E).

Fluorescence-related parameters were assessed following the methodology outlined by Flexas et al. (2002). PSII photochemical efficiency (PhiPS2) and electron transport rate (ETR) were measured concurrently with gas exchange measurements. Maximum quantum efficiency of PSII (Fv/Fm) was determined after a 4-h dark adaptation period. Non-photochemical quenching (NPQ) was computed as described by Flexas et al. (2002). To ensure the equipment's proper functioning and confirm the optimal photosynthetic status of control plants at the experiment’s outset, the ratio of electron transport rate to assimilation rate (ETR/An) was monitored, following Flexas et al. (2002) and Perera-Castro and Flexas (2023).

### Antioxidant measurements

After two months of salinity exposure and subsequent physiological measurements, leaf samples were collected from each treatment group (N = 10) for both species. Samples were immediately immersed in liquid nitrogen for rapid cold storage and later maintained at − 80 °C until biochemical analysis. Leaf samples were homogenized in 50 mM Tris HCl buffer containing 1 mM ethylenediaminetetraacetic acid (EDTA) at pH 7.5, using a weight-to-volume ratio of 1:5. Homogenization was carried out under cold conditions using an ULTRA-TURRAX® Disperser (IKA). The homogenized samples were then centrifuged at 10,000 × g and 4 °C for 10 min to remove cell debris from the supernatant, which was subsequently re-stored at − 80 °C until biochemical assays were performed. Enzyme activities were determined using a Shimadzu UV-2100 spectrophotometer at 25 °C, while lipid peroxidation was assessed using a Bio-Tek PowerWave XS microplate spectrophotometer. Total protein content per sample was measured by the Biorad® colorimetric kit, using bovine serum albumin (BSA) as a standard, and all biochemical measurements were normalised to per mg protein.

Catalase (CAT) (EC 1.11.1.6) activity was determined following the method described by Aebi (1984). This involved monitoring the decomposition of H_2_O_2_ in a 50 mM phosphate buffer at pH 7.0 by measuring the decrease in absorbance at 240 nm. CAT activity is expressed as mK(s^−1^)/mg protein. Superoxide dismutase (SOD) (EC 1.15.1.1) activity was assessed based on the inhibition of cytochrome C reduction by the superoxide anion generated via the xanthine oxidase/hypoxanthine system, according to Flohé and Otting (1984). The reaction was conducted in a 50 mM potassium phosphate buffer containing 0.1 mM EDTA at pH 7.8, and the absorbance was measured at 550 nm using an absorption coefficient of 28.1 mM^−1^ cm^−1^. SOD activity is reported as pKat/mg protein. Glutathione reductase (GRd) (EC 1.8.1.7) activity was determined by monitoring the oxidation of NADPH (9.6 mM) at 340 nm using oxidized glutathione as the substrate, following the method of Goldberg and Spooner (1984). An absorption coefficient of 6.22 nM^−1^ cm^−1^ was used, and GRd activity is presented as nKat/mg protein. Glutathione peroxidase (GPx) (EC 1.11.1.9) activity was measured using a modification of the Flohé and Gunzler (1984) method. The reaction utilized H_2_O_2_ as the substrate, with GRd as the enzymatic indicator and NADPH as the non-enzymatic indicator, supplemented with NaN_3_ to inhibit catalase. An absorption coefficient of 6.22 nM^−1^ cm^−1^ was applied. GPx activity was measured at 340 nm and is expressed as nKat/mg protein.

### Lipid peroxidation assay

Malondialdehyde (MDA) levels were utilized as an indicator of lipid peroxidation and oxidative damage, following the methodology described by Capó et al. (2020). MDA concentration was measured using a colorimetric assay, where MDA reacts with a reagent to form a stable chromophore with maximum absorbance at 586 nm. To conduct the assay, samples were treated with N-methyl-2-phenindole (10.3 mM) in acetonitrile:methanol (3:1) solution. Subsequently, 12 N HCl was added, and the samples were incubated at 45 °C for 1 h. MDA of known concentration (MAK085-1KT, Sigma-Aldrich) was used as a standard for calibration, and measurements were taken at 586 nm. Results are expressed in nmols/mg protein.

### Statistical analysis

Reproductive traits of both species were compared among treatments to evaluate the effect of seawater concentration. The effect of salinity exposure on phenology was evaluated, in the case of *P. maritimum*, modeling the number of flowers in anthesis against the date of the beginning of the experiment using Generalized Linear models (Binomial family, link logit). Similarly, in *E. maritimum* the effect of seawater concentration and date was modeled against the number of capitula in anthesis using Linear models (Gaussian family, link identity). In all the analysis, model selection was carried out using the Akaike Information Criterion (AIC). At inflorescence level, length, width, and the number of reproductive units were modeled against seawater concentration and species, considering the potential interaction among variables. For length and width, Generalized Linear models (family Gaussian, link inverse; Amin et al., 2016) were used, while for the number of reproductive units GLMs (family Poisson, link inverse) were used. At the reproductive unit level, length and width were modelled against seawater concentration and species using GLMs (family Gaussian, link = 1/mu^2; Kinat et al., 2020). Finally, the effect of seawater concentration on Fruit Set and Seed Set was modeled using GLMs (Family binomial, link logit). To evaluate, the effect of seawater watering on photosynthetic parameters, stomatal conductance (gs), intercellular CO_2_ (Ci), assimilation rate (An), and electron transport rate (ETR) were modeled considering the seawater concentration and the species as explanatory variables. For this purpose, GLMs (family Gaussian, link log) and Generalized Linear Mixed models (GLMMs) were used, using in this last case the plant as a random factor. On the other hand, fluorescence metrics (NPQ and Fv-Fm) were also considered and modeled similarly using LM and LMM (family Gaussian, link log). Finally, the variation in biochemical indicators was evaluated considering the levels of CAT, SOD, GPX, GRd and MDA in the different seawater treatments in both species and were modeled using LMs and LMMs (Family Gaussian, link identity).

All the analyses were carried out in R (R Core Team, 2022). Statistical significance of models was evaluated using anova against null models. Statistical significance of factors was evaluated using Analysis of variance in LMs and Analysis of deviance in GLMs. When applicable, differences were assessed using the Kruskal–Wallis test (McKight and Najab, 2010). Differences among treatments were analyzed using the Tukey Honest Significant Difference (HSD) test (Abdi and Williams, 2010) or the Dunn test (Dinno, 2017) when necessary. Explained variance/deviance was evaluated calculating the proportion of variance explained by the models compared with de variance-deviance of null models. Final models were plotted against observed data using ggplot2.

## Results

### Reproductive measurements

**Fig. 2:**
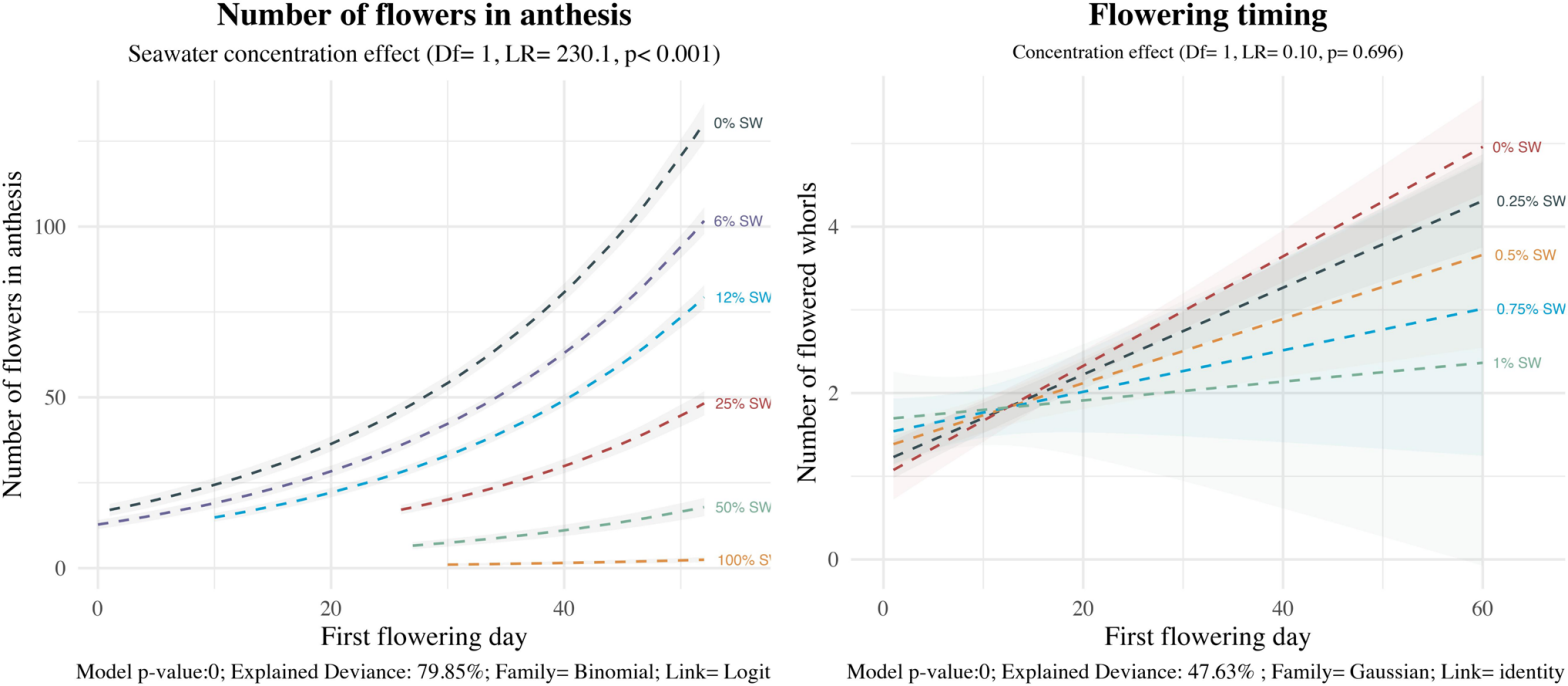
Effect of salinity concentration on the cumulative number of flowers (*P. maritimum*) and whorls (*E. maritimum*) during the experiment. The dashed line indicates the prediction of the model, while colored area indicates the model standard error. Different colors are used to indicate the different sea-water concentrations. Analysis of deviance results are indicated below each species. In caption the model p-value, the explained deviance and the family of the model are indicated

Phenology response to seawater treatments was significantly altered in *E. maritimum* being flowering slightly delayed between treatments. *P. maritimum* showed shorter flowering time with flowering being strongly delayed for high salinity treatment (25%SW onwards) (Fig. 2). For the latter, flowering period was reduced almost by 50% compared to low salinity treatments.

**Fig. 3:**
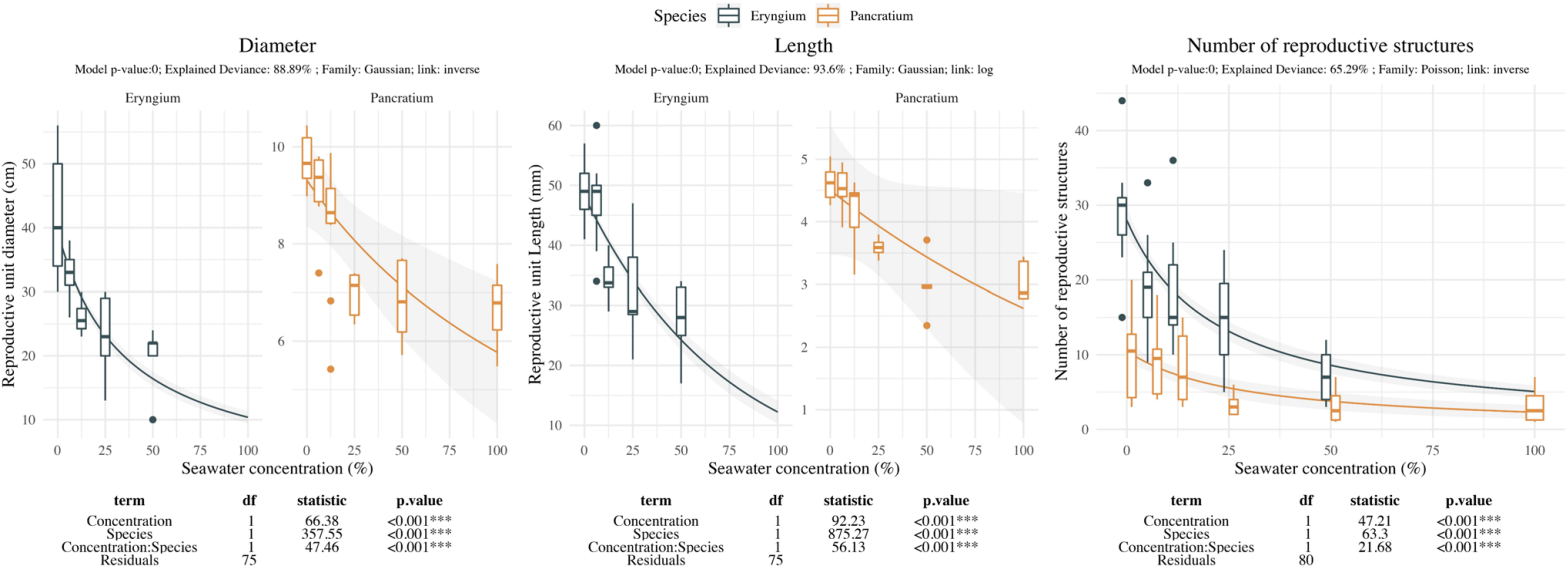
Effect of salinity concentration on inflorescence related traits. Boxplots are used to indicate the median and first to fourth quartiles. The solid line indicates the prediction of the model, while grey area indicates the model standard error. Different colors are used to ease species comparison (blue for *E. maritimum* and yellow for *P. maritimum*). Analysis of deviance results are indicated below each plot. For each response variable (Diameter, Length and Number of reproductive structures) the model *p-*value, the explained deviance and the family of the model are indicated

Seawater influence on inflorescence traits showed negative effects on both species, with decreased size (Diameter and Length) and number of reproductive units (Flowers for P. maritimum and Whorls in *E. maritimum*; Fig. 3). *E. maritimum* displayed higher decrease at 12.5%SW but showed steady response at moderate-high salinity levels (25%SW) decreasing in size but maintaining high inflorescence production. *P. maritimum* showed similar size until 12.5%SW decreasing abruptly both in number of flowers and size at 25%SW and further levels. Both species showed strong effect at 50%SW, and null flowering in *E. maritimum* and anecdotic flower production in *P. maritimum* at 100%SW.

**Fig. 4:**
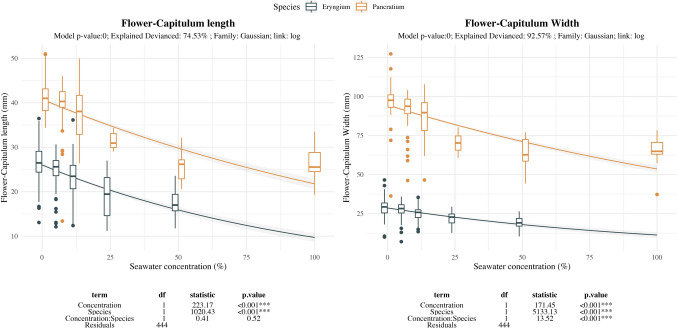
Effect of salinity concentration on flower (*P. maritimum*) and capitulum (*E. maritimum*) size. Boxplots are used to indicate the median and first to fourth quartiles. The solid line indicates the prediction of the model, while grey area indicates the model standard error. Different colors are used to ease species comparison (blue for *E. maritimum* and yellow for *P. maritimum*). Analysis of deviance results are indicated below each plot. For each response variable (Diameter, Length and Number of reproductive structures) the model *p-*value, the explained deviance and the family of the model are indicated

**Fig. 5:**
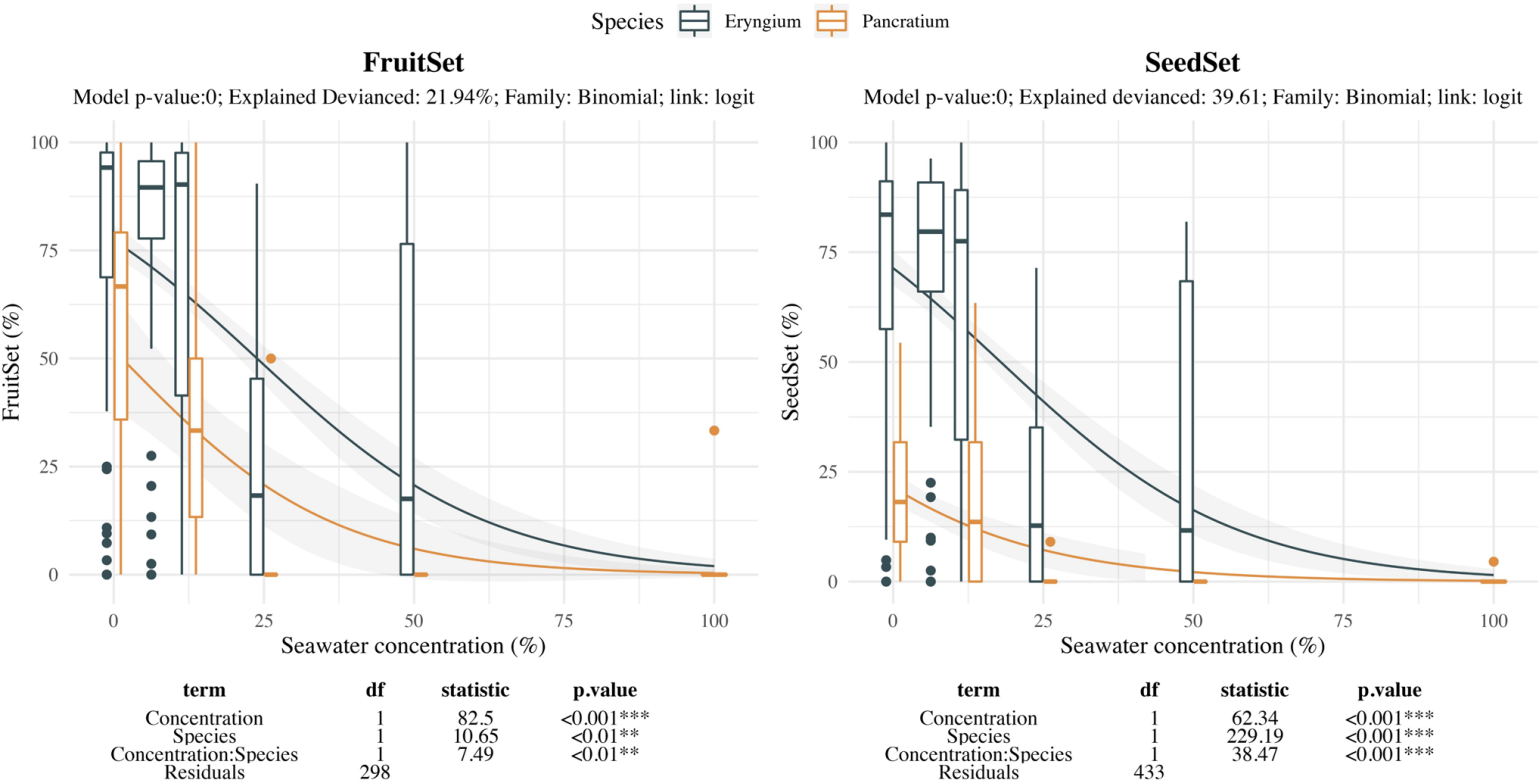
Effect of salinity concentration on fruit and seed production. Boxplots are used to indicate the median and first to fourth quartiles. The solid line indicates the prediction of the model, while grey area indicates the model standard error. Different colors are used to ease species comparison (blue for *E. maritimum* and yellow for *P. maritimum*). Analysis of deviance results are indicated below each plot. For each response variable (Fruit and Seed Set) the model *p-*value, the explained deviance and the family of the model are indicated

Seawater effect on reproductive units showed size constriction mainly at 25%SW for both species, being barely affected at lower salinity treatments (Fig. 4). Fertility related traits showed strong decrease with seawater treatment but with differing intensity depending on the species (Fig. 5). Fruit-set remained similar until 12.5%SW for *E. maritimum* while *P. maritimum* already decreased. Fruit setting at further levels was strongly reduced in *E. maritimum* and became null in *P maritimum*. Seed-set shows similar pattern for both species, with steady values until 12.5%SW and further strong decrease in the remaining treatments.

### Gas exchange and fluorescence measurements

**Fig. 6:**
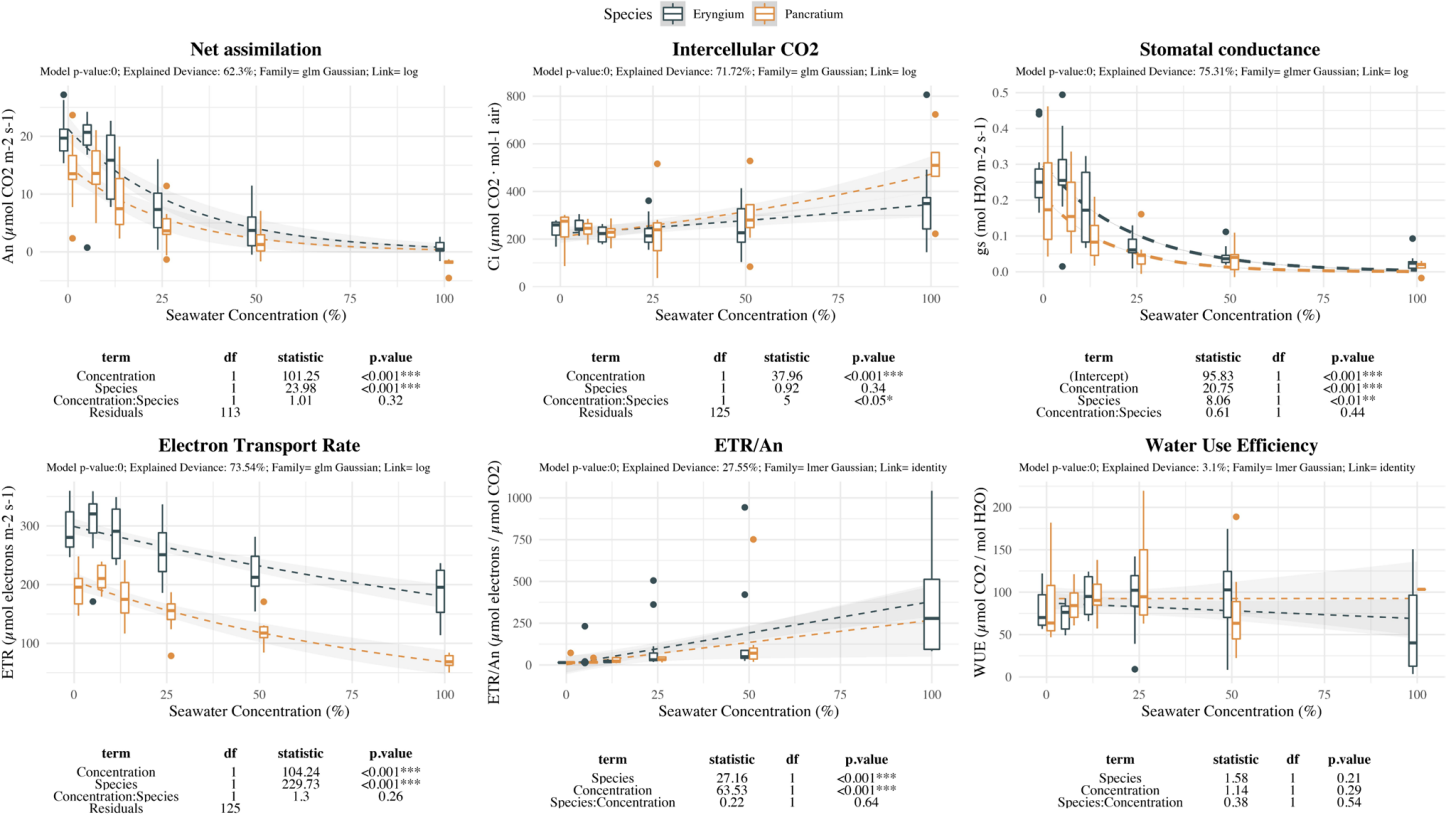
Effect of salinity concentration on photosynthetic plant response. Boxplots are used to indicate the median and first to fourth quartiles. The dashed line indicates the prediction of the model, while grey area indicates the model standard error. Different colors are used to ease species comparison (blue for *E. maritimum* and yellow for *P. maritimum*). Analysis of deviance results are indicated below each plot. For each photosynthetic indicator the model *p-*value, the explained deviance and the family of the model are indicated

Seawater effect on gas exchange measurements shows similar response for both species being only noticeable differences in the Ci pattern of response (Fig. 6). Both taxa displayed small variations on stomatal and assimilation rate until 1/4SW. Strong decrease was appreciated mainly at high salinity levels (50%SW and full SW). Ci showed a steady pattern with a small increase at full-SW for *E. maritimum*, while *P. maritimum* showed significant increase starting at 50%SW level. *P. maritimum* shows small increase at 12.5%SW and abruptly decreased for the following treatments. ETR/*An* ratio increased in both species starting at 50%SW and being maximum at 100%SW treatment in *E. maritimum*. For *P. maritimum* similar pattern can be argued, but negative assimilation rate values prevented to include 100%SW treatment.

**Fig. 7:**
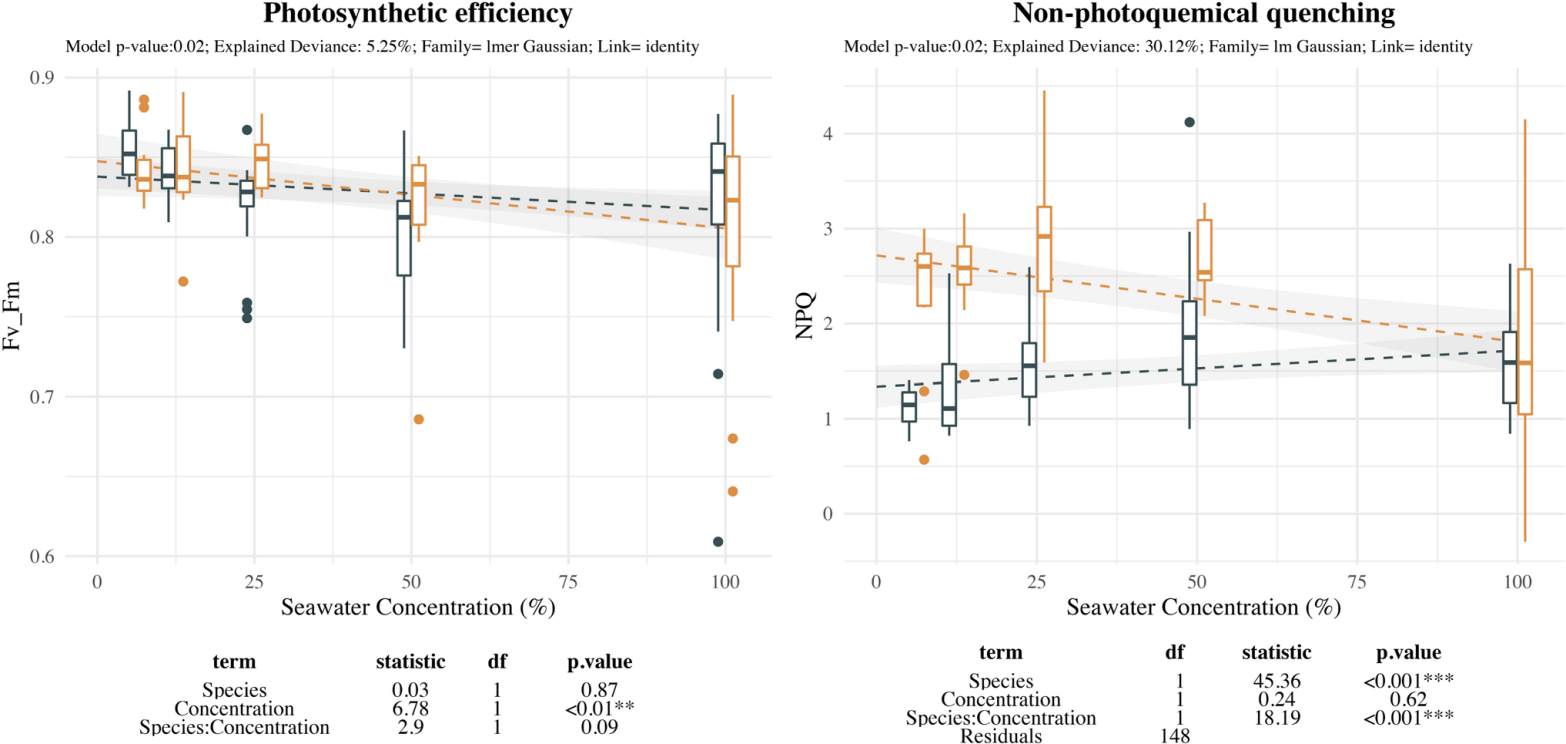
Effect of salinity concentration on fluorescence measurements. Boxplots are used to indicate the median and first to fourth quartiles. The dashed line indicates the prediction of the model, while grey area indicates the model standard error. Different colors are used to ease species comparison (blue for *E. maritimum* and yellow for *P. maritimum*). Analysis of deviance results are indicated below each plot. For each response variable (Diameter, Length and Number of reproductive structures) the model *p-*value, the explained deviance and the family of the model are indicated

Fluorescence related parameters showed contrasting response for NPQ levels between both species and slight effect of salinity concentration for Fv/Fm (Fig. 7). Fv/Fm levels maintained stable values being slightly decreased with salinity. NPQ increased with salinity in *E. maritimum* starting at 12.5%SW and remained high and stable at further levels. In contrast *P. maritimum* showed stable values at low salinity levels decreasing at higher salinity levels.

### Antioxidant enzyme activities and lipid peroxidation assay

**Fig. 8:**
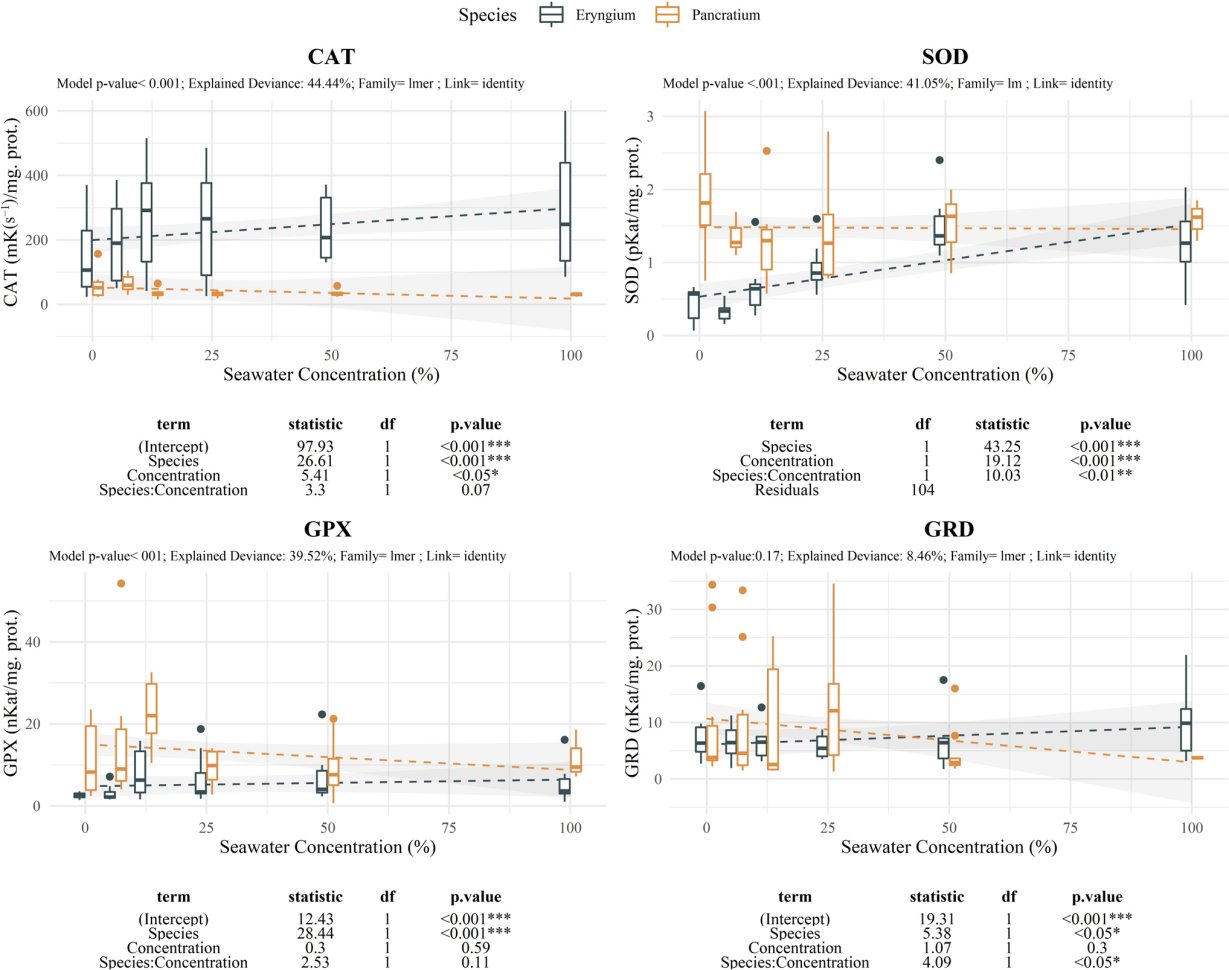
Effect of salinity concentration on key oxidate stress enzymes. Boxplots are used to indicate the median and first to fourth quartiles. The dashed line indicates the prediction of the model, while grey area indicates the model standard error. Different colors are used to ease species comparison (blue for *E. maritimum* and yellow for *P. maritimum*). Analysis of deviance results are indicated below each plot. For each enzyme the model *p-*value, the explained deviance and the family of the model are indicated

Antioxidant response showed variable patterns depending on the species and the antioxidant enzyme (Fig. 8). *E. maritimum* showed active response for SOD and CAT enzyme activity, starting at 12.5%SW and 25%SW onwards. Glutathione relates enzymes were less responsive with activation at 12.5%SW and further steady activity for GPx, and activation only at 100%SW for GRd. *P. maritimum* maintained high activity restricted to low-stress related treatments (C—12.5%SW) for SOD, CAT and GPx, and activation extended to 25%SW in GRd. Null changes in the activities were observed at further levels.

**Fig. 9:**
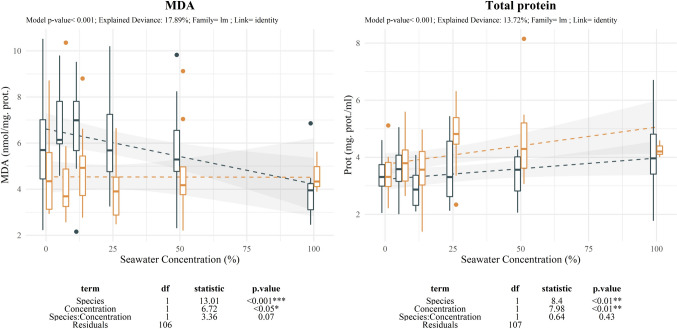
Effect of salinity concentration on Malondialdehyde (MDA) expression and Total protein content. Boxplots are used to indicate the median and first to fourth quartiles. The dashed line indicates the prediction of the model, while grey area indicates the model standard error. Different colors are used to ease species comparison (blue for *E. maritimum* and yellow for *P. maritimum*). Analysis of deviance results are indicated below each plot. For each response variable the model *p-*value, the explained deviance and the family of the model are indicated

MDA displayed contrasting patterns of variation between both taxa however no significant interaction was observed (Fig. 9). *E. maritimum* showed small non-significant increase at low levels, and decreased levels at further moderate to high salinity treatments (25%SW onwards). *P. maritimum* displayed steady levels being MDA unrelated to salinity stress treatment. However, MDA levels without considering protein content showed increasing values with salinity for the latter. Protein content in *P. maritimum* also increased with treatment.

## Discussion

Salinity is considered among the main stress sources for coastal species (Cozzolino et al. 2017). Among coastal taxa, geophytes have been regarded of interest since they colonize different plant communities and represent examples of cycle adjustment to the harsh conditions (Juan-Vicedo et al. 2021). The aim of this study was to approach the salinity response of two geophytes of Mediterranean and Atlantic coasts which have been indicated as strong halotolerant, and if their use could be suitable as gardening species in coastal areas. Overall, our data supports response in agreement with some degree of salinity tolerance but discards further tolerance definitions such as halophyte for both taxa. Inflorescence production is resilient regarding salinity which enables to consider both species for ornamental purposes.

### Physiological and antioxidant response

Physiological response shows overall small effects on gas exchange parameters with low (12.5%SW) and moderate (25%SW) salinity, followed by a noticeable depleting effect at further levels. Steady physiological response to maintain assimilation rate under field conditions has been previously indicated in both species by Bouchemal et al (2022). However, steady gas exchange at moderate salinity levels (25%SW) concurs with the starting point of antioxidant and fluorescence response to salinity. Only for *P. maritimum*, small increased Ci values at moderate salinity levels imply adjustments affecting water relations. Stomatal adjustment is known to be tidily regulated and highly responsive to water status (Munns et al., 2011; Koyro et al. 2013). *E. maritimum* is known to display leaf micromorphological traits which allow maintaining high assimilation rates with relatively low water loss (Ivanova et al. 2015). In the case of *P. maritimum*, similar traits associated with enlarged leaf thickness allow to maintain a high WUE (Perrone et al. 2015). Our results seem to support slight but higher WUE for *E. maritimum* at moderate salinity. In contrast, *P. maritimum* seems to deal with earlier stomatal and metabolic limitations. Water relations stability may also relate to specific mechanisms countering osmotic stress. Use of osmoptroectants, such as proline, has been indicated for *P. maritimum* under salt stress (Kedhr et al. 2003), while for *E. maritimum* stomatal and morphological adjustments have been considered more relevant (Boucehmal et al., 2022). Carfagna et al. (2021) found increased micronutrients (Zn and Mn) and stable K and Ca levels in leaves of *P. maritimum* exposed to salinity. Considering the competitive nature of sodium with potassium, the stable levels of potassium indicate active absorption and translocation by the root system (So et al. 2022), a process that could also occur in *E. maritimum*.

Salinity significantly affects both *E. maritimum* and *P. maritimum*, particularly at 50% and 100%SW concentrations, as evidenced by reduced fluorescence parameters Fv/Fm and increased ETR/An ratio. These changes indicate potential damage to PSII from ROS overproduction (Geissler et al. 2015; Arora et al. 2016*). E. maritimum* responds with increased NPQ, suggesting active thermal dissipation to mitigate ROS production (Galmés et al. 2007). In contrast, *P. maritimum* shows reduced heat dissipation under stress, indicating a divergent response mechanism. Antioxidant enzyme activities also exhibit contrasting patterns. *E. maritimum* displays increased superoxide dismutase (SOD) and catalase (CAT) activities, consistent with effective ROS scavenging as described in other saline-tolerant species (Bose et al. 2014; Leung et al., 2018). Despite contradicting previous data reported under field conditions which possibly relate to unstressed plants (Bouchemal et al. 2022), recent studies highlight active antioxidant capacity and ROS scavenging compounds in *E. maritimum* rhizome extracts (Cortés-Fernández et al. 2023). Activation of the glutathione system further supports *E. maritimum* response long term salinity stress (Hasanuzzaman et al. 2012). In contrast, *P. maritimum* exhibits peak enzymatic activity at lower stress levels (notably at 6.25% SW), declining with increased salinity. This contrasts with expected findings and previous reports of active antioxidant responses in similar conditions (Abogadallah 2011; Burcu et al., 2013; Carfagna et al. 2021; Khedr et al. 2003). Previous studies highlighted interference by secondary metabolites like phenolic compounds on enzymatic antioxidant measurements, such as catalase (Khataee et al. 2022). However, the consistent activity patterns observed during our measurements suggest minimal interference, making this explanation less likely. Another explanation for antioxidant enzyme activity loss has been related to leaf senescence due to stress (Trivellini et al. 2017). This idea seems to gain support if NPQ decrease is considered, which has also been related to leaf senescence (Juvany et al. 2013). Previous studies in *P. maritimum* on enzymatic response have mainly focused on short term salinity stress on juvenile stages being long-term exposure less comprehended. Long-term response has been indicated both for *E. maritimum* and *P. maritimum* to be less related to enzymatic mechanism (Bouchemal et al. 2022) but rather with other non-enzymatic antioxidant mechanism rich in both species (Elmas et al., 2017). The present results may represent two stages of salt response, being *E. maritimum* response concomitant to early stages of stress while *P. maritimum* response with late senescent stages.

The salinity response in both species shows strong tolerance and reduced cell damage, likely explaining their steady physiological performance at moderate salinity levels. MDA, an indicator of cell membrane damage, is commonly used to assess salinity stress (Hernández and Almansa 2002; Gil et al. 2020). Previous studies showed increased MDA levels in *P. maritimum* under drought and salt stress (Abogadallah 2011; Burcu et al., 2013) and in senescent leaves (Djanaguiraman and Prasad 2010). However, our results show steady MDA content with salinity. Increased protein content in salt-affected *P. maritimum* has been linked to stress conditions (Burcu et al. 2013), possibly being the cause of distort MDA levels in this study. *For E. maritimum*, MDA levels decrease from 25%SW towards higher salinity. A similar negative correlation with salinity has been described by Kumar et al. (2021) and related to activation of PSII core proteins and Rubisco. Comparable responses are seen in halophytes like *Cakile maritima* (Ksouri et al. 2007; Ellouzi et al. 2011; Amor et al. 2006), *Crithmum maritimum* (Amor et al., 2005), and *Salicornia europaea* (Ghanem et al. 2021). MDA reduction over time suggests mechanisms like chlorophyll loss (Tounketi et al., 2011) or physiological adjustments (Ozturk et al. 2012). Overall Boucehmal et al. (2022) already indicates the presence of large amounts of fatty acids which provide decreased membrane permeability and higher resistance to salinity.

Physiological and biochemical studies highlight the complexity of plant tolerance to salinity in both taxa, with an enzymatic antioxidant response in *E. maritimum* and a presumed higher relevance of non-enzymatic antioxidants in *P. maritimum* (de Felice et al., 2013; Cortés-Fernández et al. 2023). Both taxa exhibit a stasis period before and during flowering, suggesting the need to identify key genes involved in salinity stress response across different phenological stages. Gene expression research has been limited for *P. maritimum* (de Felice et al., 2013) and *E. maritimum* (Cortés-Fernández et al. 2023). Further studies should deepen in the expression of genes encoding for the synthesis of metabolites associated with osmotic adjustments, such as proline (Cerrato et al. 2022). Low salt accumulation in the rhizome and bulb is likely due to active ion transporters in the roots and salt compartmentalization within these organs (So et al. 2022). Research should focus on gene expression related to ion and sugar transporters in the root system and examine phytohormones with regulatory functions produced during stress, along with ROS scavenging enzymes and non-enzymatic antioxidants in the bulb, rhizome, and leaves.

### Reproductive response and ornamental interest

Reproductive biology has been previously studied for *E. maritimum* (Cortés-Fernández et al. 2022a) and *P. maritimum* (Medrano, 1999), but salinity response has been examined only in *E. maritimum* (Cortés-Fernández et al. 2022a, b). Both species exhibit nutrient shortage patterns where inflorescence size, reproductive units (capitula or flowers), and fruit and seed production are compromised. Reproductive decline begins at 12.5% SW and is significantly affected at 25% SW and higher salinity levels. These results align with physiological and biochemical responses, indicating high resilience to salinity. As previously discussed, both species cannot be considered halophytic since their reproductive cycle is severely compromised under high salinity stress (Yuan et al. 2019; Cortés-Fernández et al. 2022a, b). For ornamental purposes, flowering appeal is maintained at 12.5%SW (9 dS/m) water irrigation and remains relevant at 25%SW (16 dS/m). Cassaniti et al. (2013) classify 8–15 dS/m as saline and 15–45 dS/m as highly saline water. Atzori et al. (2019) define species tolerating 10–30% seawater irrigation (8–18 dS/m) as suitable for medium saline conditions. Previous studies consider species displaying low damage at 7–11 dS/m as salt-tolerant (Shillo et al. 2002; Niu and Rodriguez 2006; Cassaniti et al. 2013; García-Caparrós et al. 2016). Both species respond favorably under salinity levels exceeding coastal field conductivity values (Cortés-Fernández et al. 2022a, b), fitting them among tolerant ornamental taxa.

Both species display differences on reproductive response to salinity. Our results show contrasting response for flowering, with strong delay in *P. maritimum* and slight to almost synchrony flowering in *E. maritimum* regardless salinity stress. Van Zandt et al. (2002) indicated flowering delay in *Iris hexagona* due to possible hormonal alterations under salinity stress. Moreover, flowering initiation has been indicated to depend on temperature and water stored in the bulb, while vegetative growth is controlled by external water supply (Dafni, 1981; Al-tardeh et al. 2008). Since salinity decreases root development and accelerates leave senescence, flowering would be delayed due to lower nutrient and water storage in the bulb (Van Zandt et al., 2002; Cassaniti et al. 2013). For *E. maritimum*, almost synchronic flowering suggests probable genetic related causes for flowering timing which are probably determined by temperature and photoperiod (Cho et al. 2016). However, field studies have pointed out phenological shifts regarding sea distance arguing microclimatic conditions interfering in flowering initiation (Cortés-Fernández et al. 2022b). *E. maritimum* flowering time variation appears unrelated to salt stress, with other factors playing a more significant role, which remain to be studied. For both taxa, gene expression related to flowering initiation seems influenced by environmental factors. Further studies on *E. maritimum* should investigate gene expression related to photoperiod and temperature, as occurs with *Crithmum maritimum* (Ventura et al. 2014), since salinity affects resource allocation rather than flowering control. In *P. maritimum*, salinity may disrupt key gene expression pathways through water relations and specific metabolite expression. Prolonged synthesis of soluble protein under stress to protect the bulb may delay flowering (Alipanah et al. 2023). Studying genetic screening, such as the examination of the NF-YB3 transcription factor in *Lilium pumilum*, offers potential insights into the connection between flowering and salinity stress (So et al. 2022).

Flowering display in *P. maritimum* and *E. maritimum* is nutrient-dependent, with both species showing decreased inflorescence and flower production over time (Medrano et al. 2000; Cortés-Fernández et al. 2022b). Salinity exacerbates this decline by reducing assimilation rates and water uptake, thus disrupting resource acquisition and allocation (García-Caparrós et al. 2016). Research on species with varying salt tolerance indicates that salt stress reduces inflorescence size and quality (Boscaiu et al. 2005; Ma et al. 2020). For the studied taxa, salt stress similarly reduces the number of reproductive units (capitula in *E. maritimum* and flowers in *P. maritimum*) and overall size. However, the impact on ornamental value differs due to variations in inflorescence structure and flowering dynamics. *E. maritimum* inflorescence integrity is more resilient, with size reduction occurring through the depletion of tertiary and further capitula, resulting in simpler but intact inflorescences. In contrast, *P. maritimum* inflorescence size remains steadier, but the number of flowers decreases. Since each flower lasts only one day, this reduction shortens the flowering display period, diminishing ornamental value (van Kleunen et al. 2018). Fruit production also shows contrasting responses to salinity*. P. maritimum* fruit production decreases at 12.5%SW levels, while *E. maritimum* maintains steady fruit production. Despite this, seed-setting efficiency remains similar in both species. The differences in fruiting responses are likely due to contrasting pollination strategies, while seed setting relates to resource allocation.

### Restoration purposes and social interest

While *P. maritimum* shows variable pollination visits and relies on hawkmoths for pollination (Eisikowitch and Galil 1971; Medrano et al. 1999), *E. maritimum* is a generalist species attracting a diverse range of pollinators (Cortés-Fernández et al. 2022b). Pollinator visits are a valuable service provided by gardens (Salisbury et al. 2017) and can be enhanced by promoting native species assemblies (Fukase and Simon, 2016; Salisbury et al. 2017). Both species offer unique pollination services, adding to the aesthetic and ecological value of gardens. The use of these species contributes to ecological services, including providing resources for native pollination communities, especially *E. maritimum*. Additionally, they support the structural development of dune systems. Restoration projects using these species have shown positive effects with high plant survival and development (Romano et al. 2022). Dune-adapted species facilitate sand accretion, preventing soil erosion and promoting deeper sandy soils (Miller et al. 2003; van Puijenbroek et al. 2017). These processes are crucial for reducing seawater influence, particularly under future climate change scenarios. Deeper sandy soils decrease salt accumulation at the surface, benefiting plant rooting systems (Olmo et al. 2019), and soil accretion creates natural barriers against storms (van Puijenbroek et al. 2017). Active restoration of Mediterranean dune systems is essential to address anthropogenic threats like coastal massification (Della Bella et al. 2021) and mitigate climate change effects through carbon sequestration (Bonito et al. 2017).

## Conclusions

*P. maritimum* and *E. maritimum* show high resilience to moderate salinity. *E. maritimum* displays a more prolonged response both physiologically and regarding antioxidant response which is combined with predictable cycle flowering. *P. maritimum* shows earlier leave senescence patterns which prompts leaf loss and flowering cycle disruption. The latter translates into delayed flowering with salinity. Both species show steady reproductive response at moderate salinity levels which allows them to be considered as ornamental species. However, morphological, and architecturally inflorescences differences amplify effects on *P. maritimum* flowering in time, while *E. maritimum* maintains prolonged flowering. Richer pollinator assemblies in *E. maritimum* add further interest of the latter as ornamental species. However, both taxa seem fitted to be used as a native ornamental alternative for Mediterranean and Atlantic coastal areas.

## Supplementary Information

Below is the link to the electronic supplementary material.

Supplementary file1 (DOC 18 KB)

Supplementary file2 (DOC 13 KB)

Supplementary file3 (DOC 19 KB)

Supplementary file4 (XLSX 10 KB)

## Data Availability

The datasets generated during and/or analysed during the current study are available from the corresponding author on reasonable request.
